# Social Media Images as an Emerging Tool to Monitor Adherence to COVID-19 Public Health Guidelines: Content Analysis

**DOI:** 10.2196/24787

**Published:** 2022-03-03

**Authors:** Sean D Young, Qingpeng Zhang, Daniel Dajun Zeng, Yongcheng Zhan, William Cumberland

**Affiliations:** 1 Department of Informatics University of California Institute for Prediction Technology University of California, Irvine Irvine, CA United States; 2 Department of Emergency Medicine University of California, Irvine Irvine, CA United States; 3 School of Data Science City University of Hong Kong Kowloon, Hong Kong Hong Kong; 4 Institute of Automation Chinese Academy of Sciences Beijing China; 5 Department of Information Systems California Polytechnic State University San Luis Obispo, CA United States; 6 Department of Biostatistics University of California, Los Angeles Los Angeles, CA United States

**Keywords:** internet, social media, health informatics, tool, monitor, adherence, COVID-19, public health, guidelines, content analysis, policy

## Abstract

**Background:**

Innovative surveillance methods are needed to assess adherence to COVID-19 recommendations, especially methods that can provide near real-time or highly geographically targeted data. Use of location-based social media image data (eg, Instagram images) is one possible approach that could be explored to address this problem.

**Objective:**

We seek to evaluate whether publicly available near real-time social media images might be used to monitor COVID-19 health policy adherence.

**Methods:**

We collected a sample of 43,487 Instagram images in New York from February 7 to April 11, 2020, from the following location hashtags: #Centralpark (n=20,937), #Brooklyn Bridge (n=14,875), and #Timesquare (n=7675). After manually reviewing images for accuracy, we counted and recorded the frequency of valid daily posts at each of these hashtag locations over time, as well as rated and counted whether the individuals in the pictures at these location hashtags were social distancing (ie, whether the individuals in the images appeared to be distanced from others vs next to or touching each other). We analyzed the number of images posted over time and the correlation between trends among hashtag locations.

**Results:**

We found a statistically significant decline in the number of posts over time across all regions, with an approximate decline of 17% across each site (*P*<.001). We found a positive correlation between hashtags (#Centralpark and #Brooklynbridge: *r*=0.40; #BrooklynBridge and #Timesquare: *r*=0.41; and #Timesquare and #Centralpark: *r*=0.33; *P*<.001 for all correlations). The logistic regression analysis showed a mild statistically significant increase in the proportion of posts over time with people appearing to be social distancing at Central Park (*P*=.004) and Brooklyn Bridge (*P*=.02) but not for Times Square (*P*=.16).

**Conclusions:**

Results suggest the potential of using location-based social media image data as a method for surveillance of COVID-19 health policy adherence. Future studies should further explore the implementation and ethical issues associated with this approach.

## Introduction

Innovative surveillance methods are needed to assess adherence to COVID-19 recommendations [1], especially methods that can provide near real-time or highly geographically targeted data [2,3]. Social media, phone mobility data, and digital tracing apps have been discussed as potential data sources to use to better understand and track COVID-19–related behaviors and policy adherence [4-8]. However, no known COVID-19 or other research has examined whether social media images posted on a location hashtag (eg, #Centralpark) might inform regional surveillance and intervention efforts. Understanding these trends in local adherence to emergency public health orders could help inform public health and clinical needs. Accordingly, in a pilot study, we evaluated whether publicly available images might be used as a low-cost, near real-time method to monitor adherence to COVID-19 health policies.

## Methods

Instagram is the most popular photo-sharing application in the United States. It allows users to take pictures of their current activities and environment, and share them with others in real time. Pictures can have location tags where users can post pictures to a specific topic thread (eg, pictures taken in Central Park, New York). Approximately 37% of US adults use Instagram, with 75% of use among those 18-24 years of age, 57% among those 25-29 years of age, 47% among those 30-49 years of age, 23% among those 50-64 years of age, and 8% among people 65 years and older [9].

We built a Python web crawler to collect Instagram images and corresponding user information and metadata. Specifically, the Beautiful Soup package was used to parse the original HTML files. The keywords, with hashtags such as #brooklynbridge, were used to identify relevant data. The images were only collected from public accounts, which led to a sample of 43,487 Instagram images in New York from February 7 to April 11, 2020, during a timeline throughout which New York COVID-19–related public health recommendations shifted from no recommendations (March 1, 2020, the first confirmed case within New York; March 5, 2020, Mayor de Blasio reports that fears should not keep New Yorkers off the subway), to heightened awareness (March 7, 2020, Governor Cuomo declares a state of emergency), to statewide stay-at-home orders for all nonessential activities, including limiting all outdoor activities with a possibility of coming into close contact with others (March 22, 2020). For example, as part of the stay-at-home order, New Yorkers were instructed that all nonessential gatherings of individuals of any size for any reason should be canceled or postponed. They were also informed that individuals should limit outdoor recreational activities to noncontact and avoid activities where they come in close contact with other people [10].

Images were only collected from location hashtags known for nonessential activities or crowded spaces where it would likely be difficult for people to socially distance (#Centralpark; n=20,937), #Brooklyn Bridge (n=14,875), and #Timesquare (n=7675). These data were collected to attempt to describe the changing response to stay-at-home orders within these locations. We excluded images that might have been posted by the same person by only retaining up to 1 image per day per username. We also excluded images if we were unable to verify location. The final sample of images were manually reviewed by 23 graduate students to attempt to visually verify that the individuals in the pictures were at the hashtag locations (eg, images posted to #Brooklynbridge were excluded if they appeared to be indoors). Intercoder reliability was assessed by having students each label a subset of the sample of pictures to determine consistency. These students then reviewed the labels to resolve inconsistencies. This process occurred on the final sample.

We counted and recorded the frequency of valid daily posts at each of these hashtag locations over time (trend in frequency of posts at each hashtag location), as well as rated and counted whether the individuals in the pictures at these location hashtags were social distancing (ie, whether the individuals in the images appeared to be distanced from others vs next to or touching each other). As an additional metric for assessing the reliability/consistency of findings, we also measured the correlation between the location hashtags in their frequency of posts over time using data from the entire sample of individuals associated with these locations. The graduate students rating the pictures were provided with guidance on how to conduct the rating and reviewed for agreement, including instructing them to estimate social distancing based on individuals in the photos not touching or being directly next to other individuals (ie, not posing with, standing, or sitting next to each other).

We used R software (R Foundation for Statistical Computing) to conduct regressions analyzing the number of images posted over time and to calculate the correlation between trends among hashtag locations (eg, the correlation between frequency of daily images posted to #Centralpark and #Brooklyn Bridge). For illustration purposes, we calculated the average number of images posted before versus after the first New York case (March 1, 2020). Logistic regression was used to estimate changes in the proportion of posts exhibiting social distancing over time. City University of Hong Kong research ethics committee (#2-25-202001-01) and the University of California, Irvine Institutional Review Board approved this study.

## Results

**Figure 1:**
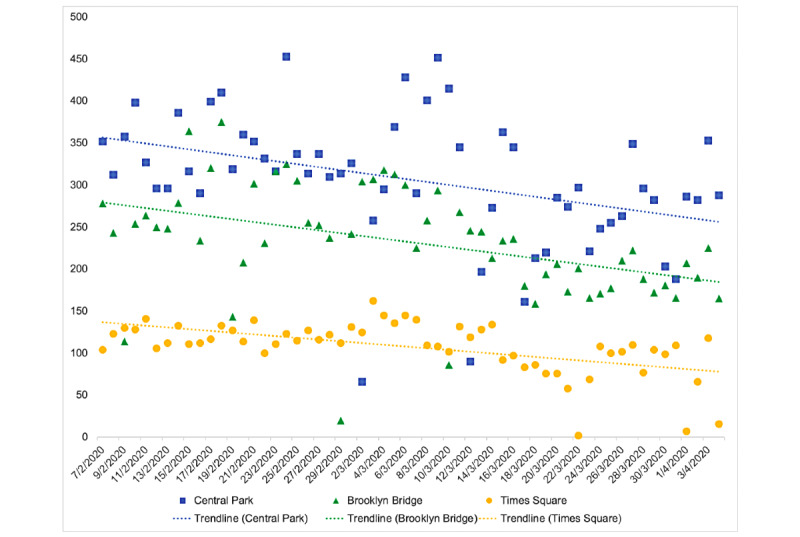
Number of images posted to the location hashtags over time, New York, 2020. Trendlines are estimated by a third-order polynomial function.

The final sample included 37,447 manually verified images: #Centralpark (n=17,761), #Brooklynbridge (n=13,459), and #Timesquare (n=6227). A total of 100 randomly selected images were reviewed by 4 of the 23 labelers to assess intercoder reliability that individuals in the pictures were at the reported hashtag locations (kappa=0.64). We found a statistically significant decline in the number of posts over time across all regions, with an approximate decline of 17% across each site (*P*<.001; Figure 1 and Table 1). We found a positive correlation between hashtags (#Centralpark and #Brooklynbridge: *r*=0.40; #BrooklynBridge and #TimesSquare: *r*=0.41; and #Timesquare and #Centralpark: *r*=0.33; *P*<.001 for all correlations). The logistic regression analysis showed a mild statistically significant increase in the proportion of posts over time with people appearing to be social distancing at Central Park (*P*=.004) and Brooklyn Bridge (*P*=.02) but not for Times Square (*P*=.16).

**Table 1:** Change in mean number of daily Instagram images for each location, New York, 2020.

Location	Number of daily posts before March 1, 2020, mean	Number of daily posts after March 1, 2020, mean (% change)	Slope	95% CI	*P* value
Central Park	342.8	282.2 (–13.7)	–1.76	–2.89 to –0.62	<.001
Brooklyn Bridge	252.9	218.3 (–17.7)	–1.66	–2.61 to –0.71	<.001
Times Square	119.8	99.2 (–17.2)	–1.03	–1.45 to –0.62	<.001

## Discussion

Results suggest that publicly available image data might be incorporated into public health surveillance methodologies as an additional tool for monitoring people’s adherence to public health guidelines, such as for the COVID-19 pandemic. We collected more than 40,000 images throughout a 10-week study period, which provided potential information about people’s locations and adherence to stay-at-home orders. Sample data were a small subset of available images, and for only 1 city, suggesting that this approach could be scaled and automated with artificial intelligence to assist with near real-time regional health surveillance. Although this is a pilot study to explore this new approach for surveillance, it provides an opportunity for future researchers to explore expanding these methods using artificial intelligence and to assess the potential cost-effectiveness of this approach.

There are a number of potential public health and emergency clinical applications of this research. First, health officials might use social media image data to better understand trends in adherence to COVID-19 prevention and other health policies. Second, using similar methods as other areas of health informatics, social media image data could be analyzed in health prediction models alongside other data sources, including case diagnoses, health services use, and demographic information, to learn how social media images might predict future COVID-19 cases within a specific region or county [11,12]. Finally, by providing data on where, when, how, and who are adhering or not adhering to health recommendations within a specific region, these data might help inform both the need for and ability to craft education and behavior change campaigns that are tailored to specific demographic or regional audiences [13,14], as well as trends in potential future local emergency department visits related to COVID-19 [15].

Although the number of posts decreased by approximately 17% throughout the study period, on average, 600 posts continued per day after issuance of stay-at-home orders, supporting the continued need for behavior change interventions. Although we found a correlation between locations, the percentage reduction in images posted was greatest for #Timesquare and least for #Centralpark. This may be because Times Square is primarily a tourist location (and tourist activity substantially diminished), while people may have continued to use Central Park to exercise.

This study was limited by a New York focus and inability to verify the exact location, time of photo, or demographic information (eg, race, sex, or home city) about the users and a biased sample of Instagram users. Future research may help to address these questions by incorporating survey data into the current types of methods. Instagram’s younger demographic [9] might help to explain the relatively small reduction in images posted after issuance of stay-at-home orders due to the common desire for independence and reduced risk perception among this age group. In addition, there are limitations with the social distancing measure (eg, it would code household members sitting together in the park having a picnic as not adhering to social distancing, when household members sitting together is appropriate, and we were unable to verify the specific number of feet individuals were from each other). We are also unable to identify why people were in these locations. As restrictions lessened over time to allow certain recreational activities, it would be helpful in future research to develop tools (eg, through artificial intelligence) that could help to identify the specific types of activities participants are doing to better understand if they were adhering to policy recommendations. Despite being publicly accessible data and social media–based public health surveillance research being supported by government agencies (eg, Centers for Disease Control and Prevention) [12,16-18], privacy and ethics-related concerns need to be further explored before implementation [19]. Relatedly, in the future, it is possible that people would intentionally choose to alter their behavior due to demand characteristics or surveillance efforts [20], including deciding to not post to certain location hashtags if they thought this method might be used for surveillance efforts. However, we believe it is unlikely that people would alter their behavior in this way as people continue to publicly share large amounts of personal health information (eg, sexual risk behaviors and drug use) on social media despite ongoing monitoring of social media and digital data [21].

As states and local health departments continue to issue public health orders, a large number of surveillance tools and approaches are needed to control the growing COVID-19 pandemic. Results from this pilot study suggest that image data might be explored and integrated with traditional epidemiology approaches to help address and better inform local health and emergency department efforts.
